# Occurrence of viable, red-pigmented haloarchaea in the plumage of captive flamingoes

**DOI:** 10.1038/srep16425

**Published:** 2015-11-10

**Authors:** Kyung June Yim, Joseph Kwon, In-Tae Cha, Kyung-Seo Oh, Hye Seon Song, Hae-Won Lee, Jin-Kyu Rhee, Eun-Ji Song, Jeong Rae Rho, Mi Lyu Seo, Jong-Soon Choi, Hak-Jong Choi, Sung-Jae Lee, Young-Do Nam, Seong Woon Roh

**Affiliations:** 1 Biological Disaster Analysis Group, Korea Basic Science Institute, Daejeon 305-806, Korea; 2 World Institute of Kimchi, Gwangju 503-360, Korea; 3 Department of Food Science and Engineering, Ewha Womans University, Seoul 120-750, Korea; 4 Research Group of Gut Microbiome, Korea Food Research Institute, Sungnam 463-746, Korea; 5 Seoul Zoo, Seoul Grand Park, Gwacheon 427-702, Korea; 6 Department of Biology and Department of Life and Nanopharmaceutical Sciences, Kyung Hee University, Seoul 130-701, Korea

## Abstract

Flamingoes (*Phoenicopterus* spp.) whose plumage displays elegant colors, inhabit warm regions close to the ocean throughout the world. The pink or reddish color of their plumage originates from carotenoids ingested from carotenoid-abundant food sources, since flamingoes are unable to synthesize these compounds de novo. In this study, viable red-colored archaeal strains classified as extremely halophilic archaea (i.e., haloarchaea) and belonging to the genera *Halococcus* and *Halogeometricum* were isolated from the plumage of flamingoes in captivity. Detailed analysis for haloarchaeal community structure in flamingo feathers based on metagenomic data identified several haloarchaeal genera and unclassified sequences of the class Halobacteria at the genus level. Carotenoid pigment analyses showed that a bacterioruberin precursor carotenoid in haloarchaea was identical to one of the pigments found in flamingo plumage. To the best of our knowledge, this is the first report of viable extremophilic archaea in avian plumage, thus contributing to our understanding of the ecology of haloarchaea. The potential influence of haloarchaea as an environmental factor determining avian plumage coloration should be investigated in further studies.

Flamingoes are pink wading birds that are found in warm regions close to the ocean on many continents. The six species of flamingo, which include *Phoenicopterus roseus* (Greater), *P. minor* (Lesser), *P. chilensis* (Chilean), *P. jamesi* (James’s), *P. andinus* (Andean), and *P. ruber* (American), inhabit salt lakes and estuaries such as brackish shallow lagoons and mud flats. The reddish color of flamingo plumage is acquired from carotenoids in aquatic organisms such as brine shrimp or cyanobacteria upon which the birds feed^1^,^2^,^3^,^4^. The birds are unable to synthesize carotenoids de novo^3^,^5^.

Extremely halophilic (i.e., salt-loving) archaea known as haloarchaea are widely distributed in hypersaline environments and samples, e.g., soda and saline lakes, solar salterns and salt-fermented foods as well as the non-freshwater habitats of flamingoes. Our previous studies have characterized haloarchaeal diversity using culture-independent^6^,^7^ and culture-dependent^8^,^9^,^10^,^11^,^12^,^13^,^14^,^15^,^16^,^17^,^18^ approaches. These extremophilic microorganisms require an NaCl concentration of at least 1.5 M for proliferation, with optimal growth observed at 3.5–4.5 M^19^,^20^, and produce red or pink carotenoid pigments, including bacterioruberin^21^,^22^,^23^,^24^,^25^.

Not only carotenoids from the diets affect the color of the avian plumage, but also other environmental factors may take part in the development of the plumage color. In this study we isolated 13 different haloarchaeal strains present in red feathers obtained from captive flamingoes, analyzed metagenomic sequences of the feathers for targeting feather-attached haloarchaea, and compared their pigments to those found in flamingo feathers and feed.

## Results

### Haloarchaeal strains isolated from flamingo feathers

**Figure 1:**
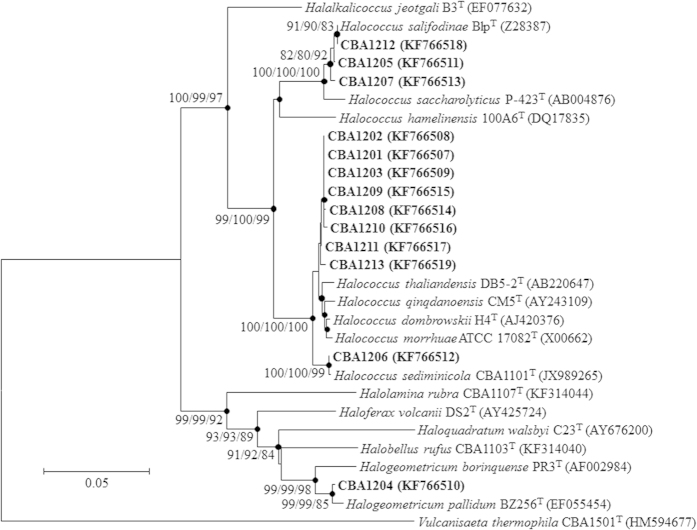
Phylogenetic tree derived from 16S rRNA gene sequences of 13 haloarchaeal isolates from flamingo feathers and phylogenetically related taxa. Bootstrap values (>70%) calculated from probabilities using neighbor-joining, minimum-evolution, and maximum likelihood algorithms are shown at the branch points. *Vulcanisaeta thermophila* CBA1501^T^ served as the outgroup. Bar, 0.05 accumulated changes per nucleotide.

A total of 13 strains of viable red-colored microorganisms were isolated from the birds’ feathers with hypersaline DBCM2 or M954 medium, but not from the artificial food consumed by flamingoes in captivity, using a culture-dependent approach, and were designated CBA1201–CBA1213 (Table 1). All of the isolated strains grew well at 37 °C and required 0–100 mM Mg^2+^. According to their phylogenetic marker (16S rRNA) gene sequences, the strains belonged to the class Halobacteria in the phylum Euryarchaeota of the domain Archaea; 12 were assigned to the genus *Halococcus* (*Hcc.*) in the family Halobacteriaceae of the order Halobacteriales and one was to the genus *Halogeometricum* (*Hgm*.) in the family Haloferacaceae of the order Haloferacales^26^. The strains formed four clusters in the phylogenetic trees constructed based on 16S rRNA sequences (Fig. 1) and were closely related to four known haloarchaea: *Hcc. salifodinae* (99.4%–100.0% similarity), *Hcc. dombrowskii* (99.1%–99.4%), *Hcc. sediminicola* (99.9%), and *Hgm. pallidum* (99.2%).

### Metagenomic analysis of microbes in the flamingo feathers

**Figure 2:**
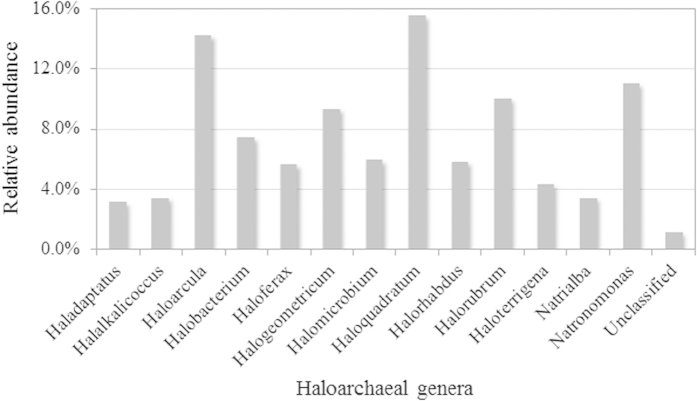
Relative abundance of haloarchaeal genera assigned to the class Halobacteria among metagenomic sequences isolated from flamingo feathers.

For a detailed investigation of archaeal community structure, of the total number of sequence reads analyzed in the metagenome dataset, 7,549 were identified as belonging to the domain Archaea, and 2,012 (26.7%) were attributed to haloarchaea. A phylogenetic classification of haloarchaeal sequences was carried out, and the 13 haloarchaeal genera (98.9% of haloarchaeal sequences) and unclassified sequences of the class Halobacteria (1.1%) were identified at the genus level (Fig. 2). Sequences belonging to the genera *Haloquadratum* (*Hqr*.), *Haloarcula* (*Har*.), *Natronomonas* (*Nmn*.), *Halorubrum* (*Hrr*.), and *Halogeometricum* (*Hgm*). collectively comprised 59.8% of haloarchaea (15.5%, 14.2%, 11.0%, 9.8%, and 9.3%, respectively), and were identified at the species level as *Hqr. walsbyi*, *Har*. *marismortui*, *Nmn*. *pharaonis*, *Hrr*. *lacusprofundi*, and *Hgm*. *borinquense*.

### Carotenoid pigment analyses

**Figure 3:**
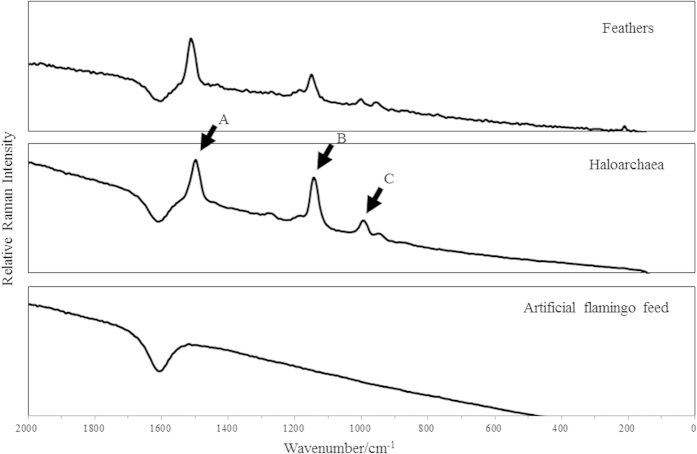
Raman spectra of flamingo feathers, isolated haloarchaeal strains, and artificial flamingo feed. Raman peaks were detected corresponding to (**A**) C=C stretching, (**B**) C–C stretching, and (**C**) C=CH bending of the pigment bacterioruberin.

**Figure 4:**
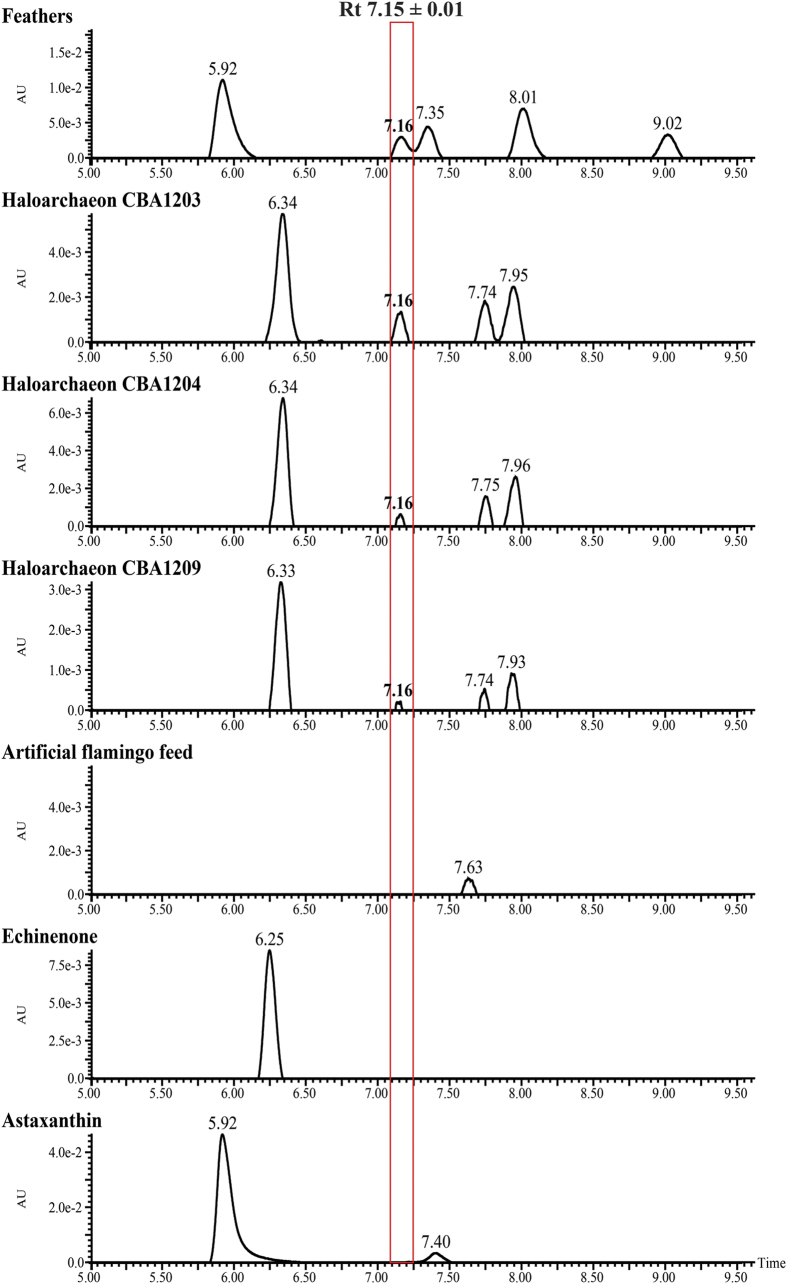
UPLC elution profiles of carotenoids extracted from flamingo feathers, three haloarchaeal strains (CBA1203, CBA1204, and CBA1209), and artificial flamingo feed, as well as echinenone and astaxanthin standards. Absorbance was measured at 450 nm.

Carotenoids from isolated haloarchaea, red flamingo feathers, and assorted artificial feed were analyzed by micro-Raman spectroscopy. Consistent Raman spectra were obtained across replicates for each type of sample, with only minor shifts within each group. The haloarchaeal strains and feathers showed similar profiles, whereas the spectrum for feed samples differed (Fig. 3). Bacterioruberin, the major carotenoid pigment responsible for the red color of haloarchaea and a biomarker for these organisms in hypersaline environments, showed two strong Raman peaks at 1505–1508 cm^−1^ and 1148–1152 cm^−1^ corresponding to C = C and C–C stretching as well as a minor peak at 996–1001 cm^−1^ corresponding to C = CH bending in bacterioruberin^21^,^22^,^27^. The major Raman bands of bacterioruberin in the haloarchaea samples (marked as A, B and C in Fig. 3) were observed in the feather samples, but not in the feed samples. Although the typical major peaks in the Raman spectra are consistent with the presence of bacterioruberin, they do not prove its presence in feather samples, since other carotenoids could exhibit similar resonances due to the low spectral resolution of Raman spectroscopy^28^. To confirm the presence of specific haloarchaeal carotenoids such as bacterioruberin or its precursors monoanhydrobacterioruberin (MABR), bisanhydrobacterioruberin (BABR), and isopentenyldehydrorhodopin (IDR)^29^ in the feather samples, carotenoid pigments extracted from feathers, haloarchaea, and feed, together with the appropriate standards, cyanobacterial carotenoid (echinenone), and microalgal carotenoid (astaxanthin) were analyzed by Ultra Performance Liquid Chromatography (UPLC). The UPLC elution profiles corresponding to the extracted carotenoids are shown in Fig. 4. The 3 randomly selected haloarchaeal strains CBA1203, CBA1204 and CBA1209 showed the same elution profile, with 4 peaks that could be assigned to bacterioruberin and its precursors, MABR, BABR, and IDR, in order of retention time, based on a previous study that identified carotenoids from a haloarchaeon^29^. The second peak obtained from the haloarchaeal strains, with a retention time of 7.15 ± 0.01 min, was identified as the bacterioruberin precursor MABR, consistent with the peak found only in the feather samples.

## Discussion

This study was based on the observation that flamingo plumage displays elegant colors similar to haloarchaeal carotenoid-based pigments. The viable red-colored haloarchaea belonging to the genera *Halococcus* and *Halogeometricum* were cultured and isolated directly from the feathers of flamingoes in captivity, using a culture-dependent approach. This is the first report of viable extremophilic archaea present in avian plumage, and provides a novel perspective on fundamental aspects of feather microbiology. Haloarchaea are currently classified into 48 genera with 177 species in the List of Prokaryotic Names with Standing in Nomenclature database^30^. Species of the *Hcc.* and *Hgm.* genera are pink- or red-pigmented, require at least 8%–15% NaCl for growth and have optimal growth at 30 °C–42 °C. *Hcc.* species are not lysed in distilled water, unlike most other haloarchaeal cells^31^. Culture-independent approach was also applied to investigate detailed haloarchaeal community structures in the flamingo feathers from which the haloarchaeal strains were isolated. A culture-independent approach that circumvents PCR amplicon sequencing biases using a next-generation sequencing platform was also applied to metagenomes obtained from flamingo feathers where the haloarchaeal strains were isolated to investigate detailed haloarchaeal community structure. Metagenomic analysis of flamingo feathers showed 13 haloarchaeal genera and unclassified sequences of the class Halobacteria at the genus level. It is interesting that no isolated *Halococcus*-related phylotypes were found in the metagenome sequences; it is possible that this discrepancy between culture-dependent and culture-independent data is caused by inefficient breakage of the thick polysaccharide layer in the cell walls of the *Halococcus* spp. using our chosen DNA extraction method.

The halotolerant *Bacillus* strain 2–9–3, isolated from a 250 million-year-old salt crystal, has been reported in 2000 as the oldest viable organism^32^. Since then, several criteria for ensuring the authenticity of isolates or DNA from novel and unusual places have been outlined in the course of the debate on the isolation of ancient DNA^33^,^34^. The presence of haloarchaea in the plumage of birds reported in this study could constitute a comparable microbiological issue since the existence of haloarchaea in bird feathers has not been previously demonstrated. The authenticity of the haloarchaeal isolates is supported by the following points regarding intra-laboratory contamination. First, the haloarchaea were isolated with extreme care and based on extensive hands-on experience with haloarchaeal cultivation. Moreover, the haloarchaea were successfully isolated only from the feather samples, but not from either pond water or soil samples in the flamingo cage. Second, none of the archaeal strains present in our laboratory are identical to the haloarchaea isolated from the feathers, as determined based on 16S rRNA gene sequences. In addition, other evidence of the presence of haloarchaea in the feathers was obtained from metagenomic sequencing of DNA extracts showing diverse haloarchaeal phylotypes.

**Figure 5:**
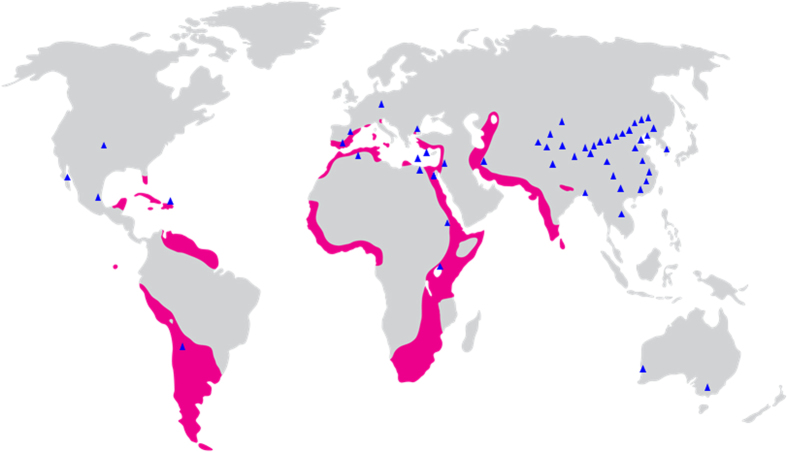
Map of the global distribution of flamingo habitats and haloarchaeal isolation sites. Flamingo habitats are indicated by a pink color and haloarchaeal sites are shown by blue triangles. The map was created using Adobe Illustrator software (Adobe Systems, Mountain View, CA, USA). The sources of haloarchaeal isolation sites can be found in Supplementary Information. The information of flamingo habitats was obtained from Wikimedia Commons (http://commons.wikimedia.org/wiki/File%3AFlamingo_range.png).

Flamingoes at the zoo were fed mainly an assortment of artificial food from which no haloarchaeal strains were isolated. In addition, the birds were not exposed to hypersaline environments since they were imported; it was therefore possible that the flamingoes had transported the haloarchaeal cells from their native hypersaline habitats. For instance, a previous study proposed that nostril salt glands of the migratory seabird *Calonectris diomedea* was a potential habitat for haloarchaea that contributed to their dispersal through the Earth’s geography^35^. It was also suggested that *Cyanobacteria* migrate between different habitats via Lesser flamingoes^36^. Flamingoes are social birds that live in colonies that can include thousands of individuals, have a behavior to rub the feathers with their heads, and drink water and wash their feathers in saline waters where haloarchaea thrive. Thus, haloarchaeal cells may adhere to feathers and be transferred between individuals by physical contact. There is some overlap in the global distribution of flamingoes and haloarchaea isolation sites (Fig. 5). The flamingoes mostly inhabit seashores or salt and soda lakes and haloarchaea were isolated from hypersaline environments such as salt lakes and solar salterns whose salt concentrations fit the growth conditions of haloarchaea. For example, *Hrr. vacuolatum*^37^,^38^ and *Natronococcus amylolyticus*^39^ were isolated from Lake Magadi, an alkaline soda lake that is also an optimal habitat for millions of flamingoes in Kenya, Africa. *Hrr. ezzemoulense*^40^ was collected from Ezzemoul, a Greater flamingo breeding site in Algeria^41^. In addition, *Halomicrobium katesii*^42^ and *Hrr. tebenquichense*^43^ were isolated from Lake Tebenquiche, the northern part of the Atacama Saltern in Chile where Andean flamingoes flock.

For verification of correlation between pigments of flamingo plumage and the isolated haloarchaea, carotenoid-based pigments from the red flamingo feathers and haloarchaea were analyzed using Raman spectroscopy and UPLC. The main skeletal C =C and C – C stretching bands and C =CH bend of the carotenoid Raman spectra confirmed that bacterioruberin and its precursors constitute the main carotenoids in haloarchaea, and the bacterioruberin precursor carotenoid identified by UPLC in haloarchaea was identical to one of the pigments in the feathers, but not to pigments in the assorted artificial flamingo feed. Plumage pigments have been characterized in swallows, bluebirds, penguins, chickens^44^,^45^, parrots^46^,^47^, and mallards^48^, and mostly consist of melanins and carotenoids^3^,^49^. While the former are synthesized independently of ingested nutrients, carotenoids—which emerged in archaea as lipophilic biochemicals reinforcing cell membranes and serving as antioxidants and confer a red, orange, or yellow color to avian feathers—are only acquired by ingestion of carotenoid feeding^1^,^3^,^5^. The pink or reddish colors of flamingo plumage originated from the carotenoids by carotenoid-abundant feed often in hypersaline lakes where is one of the haloarchaeal habitats. Carotenoid-based plumage coloration occurs through a mechanism involving carotenoid transport to blood and uptake by follicle cells in feathers^1^,^50^,^51^,^52^. Analysis of the major carotenoid pigments of haloarchaea^21^,^22^,^23^,^24^,^25^,^29^ revealed that the isoprenoid-derived carotenoids, bacterioruberin and its precursors, are present in the haloarchaea membrane, giving rise to the characteristic bright pinkish-red color.

In this study, haloarchaeal community structures in red flamingo feathers were analyzed by culture-dependent and culture-independent approaches. The unusual existence of the extremophilic archaea in the avian plumage can provide fundamental insight to the feather microbiologist, as well as birder and ornithologist. The bacterioruberin precursor responsible for the red coloration of haloarchaea was consistent with one of the carotenoid pigments found in flamingo feathers. The carotenoid-based plumage coloration of the molting flamingoes that have been mostly fed the restricted diet without the carotenoid pigments at the zoo is not explained satisfactorily with the known coloration mechanisms. The coincidence of the carotenoid-based pigments in the haloarchaeal cells and the avian feathers suggests that haloarchaea may be considered as an environmental factor affecting the plumage coloration. The biological significances of the findings as well as the possible mechanisms for carotenoid-based plumage coloration by microorganisms need to be investigated in future studies.

## Methods

### Sampling of flamingo feathers and feed

Flamingo feathers and feed were obtained from the Seoul Zoo of Grand Park in the Republic of Korea on July 5, 2013. Chilean (*Phoenicopterus chilensis*), Lesser (*P. minor*), Greater (*P. roseus*), and American (*P. ruber*) flamingoes were raised together in a cage and were fed an assorted, commercial, artificial feed (45% carbohydrate, 20% protein, 5.5% lipid, 8% mineral, and 10% water) along with shrimp irregularly (for a while), not always. Most of the flamingoes were imported from Cuba in 2004 and still remain in the same cage, and there were no records of the countries of origin for the remaining individuals. Flamingo feathers were randomly collected from the birds and these along with the feed were transported directly to the laboratory. The animal breeding protocols were approved by the institutional committee of the Seoul Zoo of Grand Park and the sampling methods were carried out in accordance with the approved guidelines.

### Isolation and characterization of haloarchaeal strains

DSMZ-German Collection of Microorganisms and Cell Cultures Medium no. 371 (M371), 372 (M372) and 954 (M954), Japan Collection of Microorganisms medium no. 788 (M788), and DBCM2^20^ were used to culture haloarchaeal strains. The pH of the media was adjusted to pH 7.0 for M372, M954, and DBCM2 and pH 9.0 for M371 and M788. Flamingo feathers (0.2–0.5 g) and feed (1.0 g) were soaked in each broth medium (30 ml) in triplicate and incubated on a shaker for 5–10 min, and 100-μl aliquots were plated onto the same medium containing 1.5% (w/v) agar. The plates were incubated at 37 °C for >2 months until pink or red colonies appeared. Colonies were transferred to fresh plates at least three times in order to obtain a pure culture. The isolated strains were cultured on various media and their growth capacity was determined. The Mg^2+^ requirement for growth was tested in M372 containing different Mg^2+^ concentrations (0, 5, 10, 20, 50, 100, 200, and 500 mM).

### Phylogenetic analysis of isolated haloarchaeal strains

The 16S rRNA gene sequences of the isolates were analyzed by colony PCR using the primer set arch20F and 1492R^53^. Purified PCR amplicons were sequenced by Sanger sequencing using arch20F, arch344F, arch520R, arch787F, 1059R, and 1492R primers. The partial 16S rRNA gene sequences were assembled using SeqMan (DNAStar, Madison, WI, USA). Nucleotide similarity values were calculated using EzTaxon-e server^54^. The 16S rRNA gene sequences of related taxa were obtained from EzTaxon-e and used for phylogenetic analysis. Sequence alignments were performed using SILVA Incremental Aligner (Bremen, Germany), taking into consideration the secondary structure of the rRNA gene^55^. Phylogenetic trees were constructed with the neighbor-joining^56^, minimum evolution^57^, and maximum likelihood^58^ algorithms based on Kimura’s two-parameter model^59^ with 1,000 randomly selected bootstrap replicates using MEGA5^60^.

### Analysis of microbial community structure in flamingo plumage

Metagenomes were extracted from flamingo feathers using the G-spin Genomic DNA Extraction kit (iNtRON Biotechnology, Seongnam, Korea) and sample libraries were constructed using the Ion Plus Fragment Library kit (Thermo Fisher Scientific, Waltham, MA, USA). Crude libraries were size-selected for 400- and 450-bp fragments with the E-Gel SizeSelect 2% agarose gel system (Thermo Fisher Scientific), and the final libraries were quantified using a Bioanalyzer 2100 (Agilent Technologies, Inc., Santa Clara, CA, USA) with high-sensitivity DNA chips. Emulsion PCR was performed using an Ion Torrent OneTouch 400 Template kit (Thermo Fisher Scientific) and libraries were sequenced using the Ion Torrent PGM sequencer (400-bp library)^61^ with the 318D sequencing chip according to the manufacturer’s instructions. Microbial community analysis was carried out using the metagenomics RAST server^62^.

### Carotenoid analysis by Raman spectroscopy

For carotenoid analysis, cells from isolated haloarchaeal strains, red flamingo feathers, and commercial feed were lyophilized, and carotenoid content was analyzed in triplicate for each sample by micro-Raman spectroscopy (Nanofinder 30; Tokyo Instruments, Tokyo, Japan) according to a previously described method^21^ with the following modifications: a 488-nm laser was used for excitation at a laser power of 2.014 mW, with a spectral resolution of 7 cm^−1^.

### Carotenoid analysis by UPLC

Carotenoids were extracted under dim light from three randomly selected haloarchaeal strains and commercial feed according to a previously described method^63^ with some modifications. Samples (0.1 g) were ground using liquid nitrogen, extracted with 3 ml of acetone, and shaken for 20 min at 60 rpm. Next, 1 ml of hexane, 3 ml water and 0.1 g NaCl were added to the acetone extract. The supernatant was transferred to a vial to remove the water soluble compounds, kept in the dark for 20 min, and then shaken for 20 min at 60 rpm. The hexane extract was evaporated with nitrogen gas, eluted with 1 ml of methanol and filtered through a 0.2 μm filter. Carotenoid extraction from red feathers was performed under dim light as follows: the feathers were ground using liquid nitrogen and the ground sample (0.2 g) was added to 2 ml of solution (methanol:water, 100:10) and 2 ml petroleum ether in a bottle. The bottle was rotated for 20 min and the upper layer was discarded. The residue was heated in boiling water for 5 min, cooled in a water bath at 37 °C, evaporated with nitrogen gas, eluted with 1 ml of methanol and filtered. Echinenone (ChromaDex, Santa Ana, CA, USA) and astaxanthin (Sigma Chemical Co., St. Louis, MO, USA) were used as carotenoid standards. All fractions were analyzed by Acquity UPLC (Waters, Wilmslow, UK). Chromatographic separation was performed at 40 °C on an Acquity UPLC BEH C18 column (2.1 mm ID × 100 mm, 1.7 μm particles; Waters). The mobile phase for the analysis method consisted of 10 mM ammonium acetate buffer (mobile phase A) and 0.1% formic acid in acetonitrile (mobile phase B). Samples were analyzed using a 15 min (total) gradient: gradient profile (% A) was set as 95% mobile phase A at 0 min, 20% A over 2 min followed by a linear increase to 100% B over 13.5 min, then an isocratic elution with 95% A between 14 and 15 min. The flow rate was set at 0.4 ml/min, and the injection volume was 5 μl. Detection was performed at 450 nm using a PDA detector (Waters).

### Data access

The GenBank/EMBL/DDBJ accession numbers for the 16S rRNA gene sequences of the isolated haloarchaeal strains are KF766507–KF766519. The metagenome sequences of the flamingo feathers have been deposited in the MG-RAST server under accession numbers 4547548.3, 4556310.3, and 4556311.3.

## Additional Information

**How to cite this article**: Yim, K. J. *et al.* Occurrence of viable, red-pigmented haloarchaea in the plumage of captive flamingoes. *Sci. Rep.* **5**, 16425; doi: 10.1038/srep16425 (2015).

## Supplementary Material

Supplementary Information

## Figures and Tables

**Table 1:** Identification of haloarchaeal strains isolated from flamingo feathers.

Strain	Closest taxon (accession no.)	16S rRNA gene similarity (%)	Minimum concentration (mM) of Mg^2+^ for growth
CBA1201	*Halococcus dombrowskii* H4^T^ (AJ420376)	99.1	10
CBA1202	*Halococcus dombrowskii* H4^T^ (AJ420376)	99.1	5
CBA1203	*Halococcus dombrowskii* H4^T^ (AJ420376)	99.1	5
CBA1204	*Halogeometricum pallidum* BZ256^T^ (HM185493)	99.2	0
CBA1205	*Halococcus salifodinae* Blp^T^ (AB004877)	99.9	100
CBA1206	*Halococcus sediminicola* CBA1101^T^ (JX989265)	99.9	0
CBA1207	*Halococcus salifodinae* Blp^T^ (AB004877)	99.4	10
CBA1208	*Halococcus dombrowskii* H4^T^ (AJ420376)	99.3	5
CBA1209	*Halococcus dombrowskii* H4^T^ (AJ420376)	99.1	5
CBA1210	*Halococcus dombrowskii* H4^T^ (AJ420376)	99.1	5
CBA1211	*Halococcus dombrowskii* H4^T^ (AJ420376)	99.4	10
CBA1212	*Halococcus salifodinae* Blp^T^ (AB004877)	100.0	20
CBA1213	*Halococcus dombrowskii* H4^T^ (AJ420376)	99.4	0

The 16S rRNA gene sequence comparisons were carried out based on sequences in the EzTaxon-e database^54^. The percent similarity between the isolated strains and known haloarchaea taxa is indicated.

## References

[b1] Hill G. E., Montgomerie R., Inouye C. Y. & Dale J. Influence of dietary carotenoids on plasma and plumage colour in the house finch: intra- and intersexual variation. Funct. Ecol. 8, 343–350 (1994).

[b2] Hill G. E., Inouye C. Y. & Montgomerie R. Dietary carotenoids predict plumage coloration in wild house finches. Proc. Biol. Sci. 269, 1119–1124 (2002). 12061954 10.1098/rspb.2002.1980 PMC1691014

[b3] Brush A. H. Metabolism of carotenoid pigments in birds. FASEB J. 4, 2969–2977 (1990). 2394316 10.1096/fasebj.4.12.2394316

[b4] McGraw K. J. Bird Coloration: Mechanisms and measurements. Vol. 1 (eds Hill G. E. & McGraw K. J.) Ch. 5, 177–242 (Harvard University Press, 2006).

[b5] Goodwin T. W. Carotenoids and reproduction. Biol. Rev. Camb. Philos. Soc. 25, 391–413 (1950). 24539201 10.1111/j.1469-185x.1950.tb00765.x

[b6] Roh S. W. *et al.* Investigation of archaeal and bacterial diversity in fermented seafood using barcoded pyrosequencing. ISME J. 4, 1–16 (2010). 19587773 10.1038/ismej.2009.83

[b7] Chang H. W. *et al.* Analysis of yeast and archaeal population dynamics in kimchi using denaturing gradient gel electrophoresis. Int. J. Food Microbiol. 126, 159–166 (2008). 18562030 10.1016/j.ijfoodmicro.2008.05.013

[b8] Cha I. T. *et al.* *Halobellus rufus* sp. nov., an extremely halophilic archaeon isolated from non-purified solar salt. Antonie van Leeuwenhoek 105, 925–932 (2014). 24609529 10.1007/s10482-014-0147-y

[b9] Kim T. Y. *et al.* *Natronomonas gomsonensis* sp. nov., isolated from a solar saltern. Antonie van Leeuwenhoek 104, 627–635 (2013). 23851717 10.1007/s10482-013-9970-9

[b10] Roh S. W. & Bae J. W. *Halorubrum cibi* sp. nov., an extremely halophilic archaeon from salt-fermented seafood. J. Microbiol. 47, 162–166 (2009). 19412599 10.1007/s12275-009-0016-y

[b11] Roh S. W., Lee M. L. & Bae J. W. *Haladaptatus cibarius* sp. nov., an extremely halophilic archaeon from seafood, and emended description of the genus *Haladaptatus*. Int. J. Syst. Evol. Microbiol. 60, 1187–1190 (2010). 19667394 10.1099/ijs.0.013037-0

[b12] Roh S. W.3 *et al.* *Haloterrigena jeotgali* sp. nov., an extremely halophilic archaeon from salt-fermented food. Int. J. Syst. Evol. Microbiol. 59, 2359–2363 (2009). 19620363 10.1099/ijs.0.008243-0

[b13] Roh S. W. *et al.* *Natronococcus jeotgali* sp. nov., a halophilic archaeon isolated from shrimp jeotgal, a traditional fermented seafood from Korea. Int. J. Syst. Evol. Microbiol. 57, 2129–2131 (2007). 17766885 10.1099/ijs.0.65120-0

[b14] Roh S. W. *et al.* *Halalkalicoccus jeotgali* sp. nov., a halophilic archaeon from shrimp jeotgal, a traditional Korean fermented seafood. Int. J. Syst. Evol. Microbiol. 57, 2296–2298 (2007). 17911300 10.1099/ijs.0.65121-0

[b15] Song H. S. *et al.* *Halapricum salinum* gen. nov., sp. nov., an extremely halophilic archaeon isolated from non-purified solar salt. Antonie van Leeuwenhoek 105, 979–986 (2014). 24677144 10.1007/s10482-014-0156-x

[b16] Yim K. J. *et al.* *Halorubrum halophilum* sp. nov., an extremely halophilic archaeon isolated from a salt-fermented seafood. Antonie van Leeuwenhoek 105, 603–612 (2014). 24442192 10.1007/s10482-014-0115-6

[b17] Yim K. J. *et al.* *Halococcus sediminicola* sp. nov., an extremely halophilic archaeon isolated from a marine sediment. Antonie van Leeuwenhoek 105, 73–79 (2014). 24132728 10.1007/s10482-013-0054-7

[b18] Cha I. T. *et al.* *Halolamina rubra* sp. nov., a haloarchaeon isolated from non-purified solar salt. Antonie van Leeuwenhoek 105, 907–914 (2014). 24633912 10.1007/s10482-014-0145-0

[b19] Oren A., Ventosa A. & Grant W. D. Proposed minimal standards for description of new taxa in the order *Halobacteriales*. Int. J. Syst. Bacteriol. 47, 233–238 (1997).

[b20] Dyall-Smith M. L. *The Halohandbook: Protocols for haloarchaeal genetics*. Available at http://www.haloarchaea.com/resources/halohandbook/Halohandbook_2009_v7.2mds.pdf. (2009) (Date of access:16/11/2012).

[b21] Jehlička J., Edwards H. G. & Oren A. Bacterioruberin and salinixanthin carotenoids of extremely halophilic Archaea and Bacteria: a Raman spectroscopic study. Spectrochim. Acta A Mol. Biomol. Spectrosc. 106, 99–103 (2013). 23376264 10.1016/j.saa.2012.12.081

[b22] Jehlička J. & Oren A. Use of a handheld Raman spectrometer for fast screening of microbial pigments in cultures of halophilic microorganisms and in microbial communities in hypersaline environments in nature. J. Raman Spectrosc. 44, 1285–1291 (2013).

[b23] Saralov A. I., Baslerov R. V. & Kuznetsov B. B. *Haloferax chudinovii* sp. nov., a halophilic archaeon from Permian potassium salt deposits. Extremophiles 17, 499–504 (2013). 23525908 10.1007/s00792-013-0534-8

[b24] Balashov S. P., Imasheva E. S. & Lanyi J. K. Induced chirality of the light-harvesting carotenoid salinixanthin and its interaction with the retinal of xanthorhodopsin. Biochemistry 45, 10998–11004 (2006). 16953586 10.1021/bi061098i PMC2528006

[b25] Saralov A. I., Baslerov R. V., Reutskikh E. M. & Kuznetsov B. B. *Halarchaeum solikamskense* sp. nov., a thermotolerant neutrophilic haloarchaeon from the foamy products of flotation enrichment of potassium minerals. Mikrobiologiia 81, 638–644 (2012). 23234075

[b26] Gupta R. S., Naushad S. & Baker S. Phylogenomic analyses and molecular signatures for the class *Halobacteria* and its two major clades: A proposal for division of the class *Halobacteria* into an emended order *Halobacteriales* and two new orders, *Haloferacales* ord. nov. and *Natrialbales* ord. nov. Int. J. Syst. Evol. Microbiol. 65, 1050–1069 (2015). 25428416 10.1099/ijs.0.070136-0

[b27] Marshall C. P. *et al.* Carotenoid analysis of halophilic archaea by resonance Raman spectroscopy. Astrobiology 7, 631–643 (2007). 17723094 10.1089/ast.2006.0097

[b28] Winters Y. D., Lowenstein T. K. & Timofeeff M. N. Identification of carotenoids in ancient salt from Death Valley, Saline Valley, and Searles Lake, California, using laser Raman spectroscopy. Astrobiology 13, 1065–1080 (2013). 24283928 10.1089/ast.2012.0952

[b29] Yatsunami R. *et al.* Identification of carotenoids from the extremely halophilic archaeon *Haloarcula japonica*. Front. Microbiol. 5, 100 (2014). 24672517 10.3389/fmicb.2014.00100 PMC3956123

[b30] Parte A. C. LPSN—list of prokaryotic names with standing in nomenclature. Nucleic Acids Res. 42, D613–616 (2014). 24243842 10.1093/nar/gkt1111 PMC3965054

[b31] Cui H. L. *et al.* *Halosarcina limi* sp. nov., a halophilic archaeon from a marine solar saltern, and emended description of the genus *Halosarcina*. Int. J. Syst. Evol. Microbiol. 60, 2462–3466 (2010). 19946053 10.1099/ijs.0.018697-0

[b32] Vreeland R. H., Rosenzweig W. D. & Powers D. W. Isolation of a 250 million-year-old halotolerant bacterium from a primary salt crystal. Nature 407, 897–900 (2000). 11057666 10.1038/35038060

[b33] Cooper A. & Poinar H. N. Ancient DNA: do it right or not at all. Science 289, 1139–1139 (2000). 10970224 10.1126/science.289.5482.1139b

[b34] Willerslev E. & Hebsgaard M. B. New evidence for 250 Ma age of halotolerant bacterium from a Permian salt crystal: Comment and reply COMMENT. Geology 33, e93–e93 (2005).

[b35] Brito-Echeverria J. *et al.* Occurrence of *Halococcus* spp. in the nostrils salt glands of the seabird *Calonectris diomedea*. Extremophiles 13, 557–565 (2009). 19363644 10.1007/s00792-009-0238-2

[b36] Dadheech P. K. *et al.* Cyanobacterial diversity in the hot spring, pelagic and benthic habitats of a tropical soda lake. FEMS Microbiol. Ecol. 85, 389–401 (2013). 23586739 10.1111/1574-6941.12128

[b37] Mwatha W. E. & Grant W. D. *Natronobacterium vacuolata* sp. nov., a haloalkaliphilic archaeon isolated from Lake Magadi, Kenya. Int. J. Syst. Bacteriol. 43, 401–404 (1993).

[b38] Kamekura M. *et al.* Diversity of alkaliphilic halobacteria: proposals for transfer of *Natronobacterium vacuolatum*, *Natronobacterium magadii*, and *Natronobacterium pharaonis* to *Halorubrum*, *Natrialba*, and *Natronomonas* gen. nov., respectively, as *Halorubrum vacuolatum* comb. nov., *Natrialba magadii* comb. nov., and *Natronomonas pharaonis* comb. nov., respectively. Int. J. Syst. Bacteriol. 47, 853–857 (1997). 9226918 10.1099/00207713-47-3-853

[b39] Kanal H., Kobayashi T., Aono R. & Kudo T. *Natronococcus amylolyticus* sp. nov., a haloalkaliphilic archaeon. Int. J. Syst. Bacteriol. 45, 762–766 (1995). 7547296 10.1099/00207713-45-4-762

[b40] Kharroub K. *et al.* *Halorubrum ezzemoulense* sp. nov., a halophilic archaeon isolated from Ezzemoul sabkha, Algeria. Int. J. Syst. Evol. Microbiol. 56, 1583–1588 (2006). 16825633 10.1099/ijs.0.64272-0

[b41] Khelifa R. *et al.* A new breeding site for the Greater Flamingo Phoenicopterus roseus in Algeria. Flamingo, Bulletin of the IUCNSSC/Wetlands International Flamingo Specialist Group, 44–47 (2009).

[b42] Kharroub K. *et al.* *Halomicrobium katesii* sp. nov., an extremely halophilic archaeon. Int. J. Syst. Evol. Microbiol. 58, 2354–2358 (2008). 18842855 10.1099/ijs.0.65662-0

[b43] Lizama C. *et al.* *Halorubrum tebenquichense* sp. nov., a novel halophilic archaeon isolated from the Atacama Saltern, Chile. Int. J. Syst. Evol. Microbiol. 52, 149–155 (2002). 11837297 10.1099/00207713-52-1-149

[b44] McGraw K. J. *et al.* You can’t judge a pigment by its color: carotenoid and melanin content of yellow and brown feathers in swallows, bluebirds, penguins, and domestic chickens. The Condor 106, 390–395 (2004).

[b45] Muroya S., Tanabe R., Nakajima I. & Chikuni K. Molecular characteristics and site specific distribution of the pigment of the silky fowl. J. Vet. Med. Sci. 62, 391–395 (2000). 10823725 10.1292/jvms.62.391

[b46] McGraw K. J. & Nogare M. C. Distribution of unique red feather pigments in parrots. Biol. Lett. 1, 38–43 (2005). 17148123 10.1098/rsbl.2004.0269 PMC1629064

[b47] Stradi R., Pini E. & Celentano G. The chemical structure of the pigments in Ara macao plumage. Comp. Biochem. Physiol. B Biochem. Mol. Biol. 130, 57–63 (2001). 11470444 10.1016/s1096-4959(01)00402-x

[b48] Haase E., Ito S. & Wakamatsu K. Influences of sex, castration, and androgens on the eumelanin and pheomelanin contents of different feathers in wild mallards. Pigment Cell Res. 8, 164–170 (1995). 7567793 10.1111/j.1600-0749.1995.tb00658.x

[b49] Hill G. E. National Geographic Bird Coloration. National Geographic (2010).

[b50] Trams E. G. Carotenoid transport in the plasma of the scarlet ibis (*Eudocimus ruber*). Comp. Biochem. Physiol. 28, 1177–1184 (1969). 5786823 10.1016/0010-406x(69)90558-1

[b51] Fox D. L. Metabolic fractionation, storage and display of carotenoid pigments by flamingoes. Comp. Biochem. Physiol. 6, 1–40 (1962). 13959019 10.1016/0010-406x(62)90040-3

[b52] Fox D. L., Hopkins T. S. & Zilversmit D. B. Blood carotenoids of the roseate spoonbill. Comp. Biochem. Physiol. 14, 641–649 (1965). 14327576 10.1016/0010-406x(65)90251-3

[b53] DeLong E. F. Archaea in coastal marine environments. Proc. Natl. Acad. Sci. USA 89, 5685–5689 (1992). 1608980 10.1073/pnas.89.12.5685 PMC49357

[b54] Kim O. S. *et al.* Introducing EzTaxon-e: a prokaryotic 16S rRNA gene sequence database with phylotypes that represent uncultured species. Int. J. Syst. Evol. Microbiol. 62, 716–721 (2012). 22140171 10.1099/ijs.0.038075-0

[b55] Pruesse E., Peplies J. & Glöckner F. O. SINA: accurate high-throughput multiple sequence alignment of ribosomal RNA genes. Bioinformatics 28, 1823–1829 (2012). 22556368 10.1093/bioinformatics/bts252 PMC3389763

[b56] Saitou N. & Nei M. The neighbor-joining method: a new method for reconstructing phylogenetic trees. Mol. Biol. Evol. 4, 406–425 (1987). 3447015 10.1093/oxfordjournals.molbev.a040454

[b57] Nei M., Kumar S. & Takahashi K. The optimization principle in phylogenetic analysis tends to give incorrect topologies when the number of nucleotides or amino acids used is small. Proc. Natl. Acad. Sci. USA 95, 12390–12397 (1998). 9770497 10.1073/pnas.95.21.12390 PMC22842

[b58] Felsenstein J. Evolutionary trees from DNA sequences: a maximum likelihood approach. J. Mol. Evol. 17, 368–376 (1981). 7288891 10.1007/BF01734359

[b59] Kimura M. A simple method for estimating evolutionary rates of base substitutions through comparative studies of nucleotide sequences. J. Mol. Evol. 16, 111–120 (1980). 7463489 10.1007/BF01731581

[b60] Tamura K. *et al.* MEGA5: molecular evolutionary genetics analysis using maximum likelihood, evolutionary distance, and maximum parsimony methods. Mol. Biol. Evol. 28, 2731–2739 (2011). 21546353 10.1093/molbev/msr121 PMC3203626

[b61] Rothberg J. M. *et al.* An integrated semiconductor device enabling non-optical genome sequencing. Nature 475, 348–352 (2011). 21776081 10.1038/nature10242

[b62] Meyer F. *et al.* The metagenomics RAST server—a public resource for the automatic phylogenetic and functional analysis of metagenomes. BMC Bioinformatics 9, 386 (2008). 18803844 10.1186/1471-2105-9-386 PMC2563014

[b63] Parisenti J. *et al.* Total carotenoid content of shrimp commercialized in Florianopolis/SC and evaluation of color preference for consumers. Alim. Nutr. Araraquara 22, 17–20 (2011).

