# Supplementary material for: Occurrence of viable, red-pigmented haloarchaea in the plumage of captive flamingoes
Source: Sci Rep. 2015 Nov 10;5:16425. doi: 10.1038/srep16425 (PMC4639753; doi:10.1038/srep16425)
Supplement: Supplementary Information [file srep16425-s1.pdf]

Supplementary Information

Occurrence of viable, red-pigmented haloarchaea in the plumage of captive flamingoes

Kyung June Yim, Joseph Kwon, In-Tae Cha, Kyung-Seo Oh, Hye Seon Song, Hae-Won Lee, Jin-Kyu Rhee, Eun-Ji Song, Jeong Rae Rho, Mi Lyu Seo, Jong-Soon Choi, Hak-Jong Choi, Sung-Jae Lee, Young-Do Nam*, and Seong Woon Roh*

List of published articles for information on haloarchaea isolation sites shown in Figure 5.

Albuquerque L, Taborda M, La Cono V, Yakimov M, da Costa MS (2012). *Natrinema salaciae* sp. nov., a halophilic archaeon isolated from the deep, hypersaline anoxic Lake Medee in the Eastern Mediterranean Sea. *Syst Appl Microbiol* **35**: 368-373.

Allen MA, Goh F, Leuko S, Echigo A, Mizuki T, Usami R *et al.* (2008). *Haloferax elongans* sp. nov. and *Haloferax mucosum* sp. nov., isolated from microbial mats from Hamelin Pool, Shark Bay, Australia. *Int J Syst Evol Microbiol* **58**: 798-802.

Amoozegar MA, Makhdoumi-Kakhki A, Shahzadeh Fazeli SA, Azarbaijani R, Ventosa A (2012). *Halopenitus persicus* gen. nov., sp. nov., an archaeon from an inland salt lake. *Int J Syst Evol Microbiol* **62**: 1932-1936.

Amoozegar MA, Makhdoumi-Kakhki A, Mehrshad M, Fazeli SA, Ventosa A (2013). *Halopenitus malekzadehii* sp. nov., an extremely halophilic archaeon isolated from a salt lake. *Int J Syst Evol Microbiol* **63**: 3232-3236.

Antunes A, Taborda M, Huber R, Moissl C, Nobre MF, da Costa MS (2008). *Halorhabdus tiamatea* sp. nov., a non-pigmented, extremely halophilic archaeon from a deep-sea, hypersaline anoxic basin of the Red Sea, and emended description of the genus *Halorhabdus*. *Int J Syst Evol Microbiol* **58**: 215-220.

Asker D, Ohta Y (2002). *Haloferax alexandrinus* sp. nov., an extremely halophilic canthaxanthin-producing archaeon from a solar saltern in Alexandria (Egypt). *Int J Syst Evol Microbiol* **52**: 729-738.

Bardavid RE, Mana L, Oren A (2007). *Haloplanus natans* gen. nov., sp. nov., an extremely halophilic, gas-vacuolate archaeon isolated from Dead Sea-Red Sea water mixtures in experimental outdoor ponds. *Int J Syst Evol Microbiol* **57**: 780-783.

Burns DG, Janssen PH, Itoh T, Kamekura M, Echigo A, Dyall-Smith ML (2010a). *Halonotius pteroides* gen. nov., sp. nov., an extremely halophilic archaeon recovered from a saltern crystallizer. *Int J Syst Evol Microbiol* **60**: 1196-1199.

Burns DG, Janssen PH, Itoh T, Minegishi H, Usami R, Kamekura M *et al.* (2010b). *Natronomonas moolapensis* sp. nov., non-alkaliphilic isolates recovered from a solar saltern crystallizer pond, and emended description of the genus *Natronomonas*. *Int J Syst Evol Microbiol* **60**: 1173-1176.

Castillo AM, Gutierrez MC, Kamekura M, Ma Y, Cowan DA, Jones BE *et al.* (2006a). *Halovivax asiaticus* gen. nov., sp. nov., a novel extremely halophilic archaeon isolated from Inner Mongolia, China. *Int J Syst Evol Microbiol* **56**: 765-770.

Castillo AM, Gutierrez MC, Kamekura M, Xue Y, Ma Y, Cowan DA *et al.* (2006b). *Natrinema ejinorense* sp. nov., isolated from a saline lake in Inner Mongolia, China. *Int J Syst Evol Microbiol* **56**: 2683-2687.

Castillo AM, Gutierrez MC, Kamekura M, Xue Y, Ma Y, Cowan DA *et al.* (2006c). *Halorubrum orientale* sp. nov., a halophilic archaeon isolated from Lake Ejinor, Inner Mongolia, China. *Int J Syst Evol Microbiol* **56**: 2559-2563.

Castillo AM, Gutierrez MC, Kamekura M, Xue Y, Ma Y, Cowan DA *et al.* (2007a). *Halovivax ruber* sp. nov., an extremely halophilic archaeon isolated from Lake Xilinhot, Inner Mongolia, China. *Int J Syst Evol Microbiol* **57**: 1024-1027.

Castillo AM, Gutierrez MC, Kamekura M, Xue Y, Ma Y, Cowan DA *et al.* (2007b). *Halorubrum ejinorense* sp. nov., isolated from Lake Ejinor, Inner Mongolia, China.

Int J Syst Evol Microbiol **57**: 2538-2542.

Cha IT, Yim KJ, Song HS, Lee HW, Hyun DW, Kim KN *et al.* (2014a). *Halolamina rubra* sp. nov., a haloarchaeon isolated from non-purified solar salt. *Antonie Van Leeuwenhoek* 105: 907-914.

Cha IT, Yim KJ, Song HS, Lee HW, Hyun DW, Kim KN *et al.* (2014b). *Halobellus rufus* sp. nov., an extremely halophilic archaeon isolated from non-purified solar salt. *Antonie Van Leeuwenhoek* **105**: 925-932.

Cha IT, Yim KJ, Song HS, Lee HW, Hyun DW, Kim KN *et al.* (2014c). *Halobellus rufus* sp. nov., an extremely halophilic archaeon isolated from non-purified solar salt. *Antonie Van Leeuwenhoek* 105: 925-932.

Corral P, Gutierrez MC, Castillo AM, Dominguez M, Lopalco P, Corcelli A *et al.* (2013). *Natronococcus roseus* sp. nov., a haloalkaliphilic archaeon from a hypersaline lake. *Int J Syst Evol Microbiol* **63**: 104-108.

Cui HL, Tohty D, Feng J, Zhou PJ, Liu SJ (2006a). *Natronorubrum aibiense* sp. nov., an extremely halophilic archaeon isolated from Aibi salt lake in Xin-Jiang, China, and emended description of the genus *Natronorubrum*. *Int J Syst Evol Microbiol* **56**: 1515-1517.

Cui HL, Tohty D, Zhou PJ, Liu SJ (2006b). *Haloterrigena longa* sp. nov. and *Haloterrigena limicola* sp. nov., extremely halophilic archaea isolated from a salt lake. *Int J Syst Evol Microbiol* **56**: 1837-1840.

Cui HL, Lin ZY, Dong Y, Zhou PJ, Liu SJ (2007a). *Halorubrum litoreum* sp. nov., an extremely halophilic archaeon from a solar saltern. *Int J Syst Evol Microbiol* **57**: 2204-2206.

Cui HL, Tohty D, Liu HC, Liu SJ, Oren A, Zhou PJ (2007b). *Natronorubrum sulfidifaciens* sp. nov., an extremely haloalkaliphilic archaeon isolated from Aiding salt lake in Xin-Jiang, China. *Int J Syst Evol Microbiol* **57**: 738-740.

Cui HL, Gao X, Li XY, Xu XW, Zhou YG, Liu HC *et al.* (2010a). *Haloplanus vescus* sp. nov., an extremely halophilic archaeon from a marine solar saltern, and emended description of the genus *Haloplanus*. *Int J Syst Evol Microbiol* **60**: 1824-1827.

Cui HL, Gao X, Li XY, Xu XW, Zhou YG, Liu HC *et al.* (2010b). *Halosarcina limi* sp. nov., a halophilic archaeon from a marine solar saltern, and emended description of the genus *Halosarcina*. *Int J Syst Evol Microbiol* **60**: 2462-3466.

Cui HL, Gao X, Sun FF, Dong Y, Xu XW, Zhou YG *et al.* (2010c). *Halogranum rubrum* gen. nov., sp. nov., a halophilic archaeon isolated from a marine solar saltern. *Int J Syst Evol Microbiol* **60**: 1366-1371.

Cui HL, Gao X, Yang X, Xu XW (2010d). *Halorussus rarus* gen. nov., sp. nov., a new member of the family *Halobacteriaceae* isolated from a marine solar saltern. *Extremophiles* **14**: 493-499.

Cui HL, Li XY, Gao X, Xu XW, Zhou YG, Liu HC *et al.* (2010e). *Halopelagius inordinatus* gen. nov., sp. nov., a new member of the family *Halobacteriaceae* isolated from a marine solar saltern. *Int J Syst Evol Microbiol* **60**: 2089-2093.

Cui HL, Sun FF, Gao X, Dong Y, Xu XW, Zhou YG *et al.* (2010f). *Haladaptatus litoreus* sp. nov., an extremely halophilic archaeon from a marine solar saltern, and emended description of the genus *Haladaptatus*. *Int J Syst Evol Microbiol* **60**: 1085-1089.

Cui HL, Gao X, Yang X, Xu XW (2011a). *Haloplanus aerogenes* sp. nov., an extremely halophilic archaeon from a marine solar saltern. *Int J Syst Evol Microbiol* **61**: 965-968.

Cui HL, Gao X, Yang X, Xu XW (2011b). *Halolamina pelagica* gen. nov., sp. nov., a new member of the family *Halobacteriaceae*. *Int J Syst Evol Microbiol* **61**: 1617-1621.

Cui HL, Yang X, Gao X, Xu XW (2011c). *Halogranum gelatinilyticum* sp. nov. and

Halogranum amylolyticum sp. nov., isolated from a marine solar saltern, and emended description of the genus *Halogranum*. *Int J Syst Evol Microbiol* **61**: 911-915.

Cui HL, Yang X, Gao X, Xu XW (2011d). *Halobellus clavatus* gen. nov., sp. nov. and *Halorientalis regularis* gen. nov., sp. nov., two new members of the family *Halobacteriaceae*. *Int J Syst Evol Microbiol* **61**: 2682-2689.

Cui HL, Yang X, Mou YZ (2011e). *Salinarchaeum laminariae* gen. nov., sp. nov.: a new member of the family *Halobacteriaceae* isolated from salted brown alga *Laminaria*. *Extremophiles* **15**: 625-631.

Cui HL, Yang X, Zhou YG, Liu HC, Zhou PJ, Dyall-Smith ML (2012). *Halobellus limi* sp. nov. and *Halobellus salinus* sp. nov., isolated from two marine solar salterns. *Int J Syst Evol Microbiol* **62**: 1307-1313.

Enache M, Itoh T, Kamekura M, Teodosiu G, Dumitru L (2007). *Haloferax prahovense* sp. nov., an extremely halophilic archaeon isolated from a Romanian salt lake. *Int J Syst Evol Microbiol* **57**: 393-397.

Fan H, Xue Y, Ma Y, Ventosa A, Grant WD (2004). *Halorubrum tibetense* sp. nov., a novel haloalkaliphilic archaeon from Lake Zabuye in Tibet, China. *Int J Syst Evol Microbiol* **54**: 1213-1216.

Feng J, Zhou P, Zhou YG, Liu SJ, Warren-Rhodes K (2005). *Halorubrum alkaliphilum* sp. nov., a novel haloalkaliphile isolated from a soda lake in Xinjiang, China. *Int J Syst Evol Microbiol* **55**: 149-152.

Goh F, Leuko S, Allen MA, Bowman JP, Kamekura M, Neilan BA *et al.* (2006). *Halococcus hamelinensis* sp. nov., a novel halophilic archaeon isolated from stromatolites in Shark Bay, Australia. *Int J Syst Evol Microbiol* **56**: 1323-1329.

Gruber C, Legat A, Pfaffenhuemer M, Radax C, Weidler G, Busse HJ *et al.* (2004). *Halobacterium noricense* sp. nov., an archaeal isolate from a bore core of an alpine Permian salt deposit, classification of *Halobacterium* sp. NRC-1 as a strain of *H. salinarum* and emended description of *H. salinarum*. *Extremophiles* **8**: 431-439.

Gutierrez MC, Castillo AM, Kamekura M, Xue Y, Ma Y, Cowan DA *et al.* (2007). *Halopiger xanaduensis* gen. nov., sp. nov., an extremely halophilic archaeon isolated from saline Lake Shangmatala in Inner Mongolia, China. *Int J Syst Evol Microbiol* **57**: 1402-1407.

Gutierrez MC, Castillo AM, Kamekura M, Ventosa A (2008a). *Haloterrigena salina* sp. nov., an extremely halophilic archaeon isolated from a salt lake. *Int J Syst Evol Microbiol* **58**: 2880-2884.

Gutierrez MC, Castillo AM, Pagaling E, Heaphy S, Kamekura M, Xue Y *et al.* (2008b). *Halorubrum kocurii* sp. nov., an archaeon isolated from a saline lake. *Int J Syst Evol Microbiol* **58**: 2031-2035.

Gutierrez MC, Castillo AM, Corral P, Minegishi H, Ventosa A (2010). *Natronorubrum sediminis* sp. nov., an archaeon isolated from a saline lake. *Int J Syst Evol Microbiol* **60**: 1802-1806.

Gutierrez MC, Castillo AM, Corral P, Kamekura M, Ventosa A (2011). *Halorubrum aquaticum* sp. nov., an archaeon isolated from hypersaline lakes. *Int J Syst Evol Microbiol* **61**: 1144-1148.

Hezayen FF, Rehm BH, Tindall BJ, Steinbuchel A (2001). Transfer of *Natrialba asiatica* B1^T to *Natrialba taiwanensis* sp. nov. and description of *Natrialba aegyptiaca* sp. nov., a novel extremely halophilic, aerobic, non-pigmented member of the Archaea from Egypt that produces extracellular poly (glutamic acid). *Int J Syst Evol Microbiol* **51**: 1133-1142.

Hezayen FF, Tindall BJ, Steinbuchel A, Rehm BH (2002). Characterization of a novel halophilic archaeon, *Halobiforma haloterrestris* gen. nov., sp. nov., and transfer of

Natronobacterium nitratireducens to *Halobiforma nitratireducens* comb. nov. *Int J Syst Evol Microbiol* **52**: 2271-2280.

Hezayen FF, Gutierrez MC, Steinbuchel A, Tindall BJ, Rehm BH (2010). *Halopiger aswanensis* sp. nov., a polymer-producing and extremely halophilic archaeon isolated from hypersaline soil. *Int J Syst Evol Microbiol* **60**: 633-637.

Hu L, Pan H, Xue Y, Ventosa A, Cowan DA, Jones BE *et al.* (2008). *Halorubrum luteum* sp. nov., isolated from Lake Chagannor, Inner Mongolia, China. *Int J Syst Evol Microbiol* **58**: 1705-1708.

Itoh T, Yamaguchi T, Zhou P, Takashina T (2005). *Natronolimnobius baerhuensis* gen. nov., sp. nov. and *Natronolimnobius innermongolicus* sp. nov., novel haloalkaliphilic archaea isolated from soda lakes in Inner Mongolia, China. *Extremophiles* **9**: 111-116.

Kanal H, Kobayashi T, Aono R, Kudo T (1995). *Natronococcus amylolyticus* sp. nov., a haloalkaliphilic archaeon. *Int J Syst Bacteriol* **45**: 762-766.

Kharroub K, Quesada T, Ferrer R, Fuentes S, Aguilera M, Boulahrouf A *et al.* (2006). *Halorubrum ezzemoulense* sp. nov., a halophilic archaeon isolated from Ezzemoul sabkha, Algeria. *Int J Syst Evol Microbiol* **56**: 1583-1588.

Kharroub K, Lizama C, Aguilera M, Boulahrouf A, Campos V, Ramos-Cormenzana A *et al.* (2008). *Halomicrobium katesii* sp. nov., an extremely halophilic archaeon. *Int J Syst Evol Microbiol* **58**: 2354-2358.

Kim KK, Lee KC, Lee JS (2011). *Halogranum salarium* sp. nov., a halophilic archaeon isolated from sea salt. *Syst Appl Microbiol* **34**: 576-580.

Kim TY, Kim SJ, Park SJ, Kim JG, Cha IT, Jung MY *et al.* (2013). *Natronomonas gomsonensis* sp. nov., isolated from a solar saltern. *Antonie Van Leeuwenhoek* **104**: 627-635.

Liu BB, Tang SK, Zhang YG, Lu XH, Li L, Cheng J *et al.* (2013). *Halalkalicoccus paucihalophilus* sp. nov., a halophilic archaeon from Lop Nur region in Xinjiang, northwest of China. *Antonie Van Leeuwenhoek* **103**: 1007-1014.

Lizama C, Monteoliva-Sanchez M, Suarez-Garcia A, Rosello-Mora R, Aguilera M, Campos V *et al.* (2002). *Halorubrum tebenquichense* sp. nov., a novel halophilic archaeon isolated from the Atacama Saltern, Chile. *Int J Syst Evol Microbiol* **52**: 149-155.

Makhdoumi-Kakhki A, Amoozegar MA, Bagheri M, Ramezani M, Ventosa A (2012a). *Haloarchaeobius iranensis* gen. nov., sp. nov., an extremely halophilic archaeon isolated from a saline lake. *Int J Syst Evol Microbiol* **62**: 1021-1026.

Makhdoumi-Kakhki A, Amoozegar MA, Ventosa A (2012b). *Halovenus aranensis* gen. nov., sp. nov., an extremely halophilic archaeon from Aran-Bidgol salt lake. *Int J Syst Evol Microbiol* **62**: 1331-1336.

Mancinelli RL, Landheim R, Sanchez-Porro C, Dornmayr-Pfaffenhuemer M, Gruber C, Legat A *et al.* (2009). *Halorubrum chaoviator* sp. nov., a haloarchaeon isolated from sea salt in Baja California, Mexico, Western Australia and Naxos, Greece. *Int J Syst Evol Microbiol* **59**: 1908-1913.

Montalvo-Rodriguez R, Vreeland RH, Oren A, Kessel M, Betancourt C, Lopez-Garriga J (1998). *Halogeometricum borinquense* gen. nov., sp. nov., a novel halophilic archaeon from Puerto Rico. *Int J Syst Bacteriol* **48**: 1305-1312.

Montalvo-Rodriguez R, Lopez-Garriga J, Vreeland RH, Oren A, Ventosa A, Kamekura M (2000). *Haloterrigena thermotolerans* sp. nov., a halophilic archaeon from Puerto Rico. *Int J Syst Evol Microbiol* **50**: 1065-1071.

Mou YZ, Qiu XX, Zhao ML, Cui HL, Oh D, Dyall-Smith ML (2012). *Halohasta litorea* gen. nov. sp. nov., and *Halohasta litchfieldiae* sp. nov., isolated from the Daliang aquaculture farm, China and from Deep Lake, Antarctica, respectively. *Extremophiles*

16: 895-901.
Mwatha WE, Grant WD (1993). *Natronobacterium vacuolata* sp. nov., a haloalkaliphilic archaeon isolated from Lake Magadi, Kenya. *Int J Syst Bacteriol* **43**: 401-404.
Nagaoka S, Minegishi H, Echigo A, Shimane Y, Kamekura M, Usami R (2011). *Halostagnicola alkaliphila* sp. nov., an alkaliphilic haloarchaeon from commercial rock salt. *Int J Syst Evol Microbiol* **61**: 1149-1152.
Oren A, Ginzburg M, Ginzburg BZ, Hochstein LI, Volcani BE (1990). *Haloarcula marismortui* (Volcani) sp. nov., nom. rev., an extremely halophilic bacterium from the Dead Sea. *Int J Syst Bacteriol* **40**: 209-210.
Oren A, Gurevich P, Gemmell RT, Teske A (1995). *Halobaculum gomorrense* gen. nov., sp. nov., a novel extremely halophilic archaeon from the Dead Sea. *Int J Syst Bacteriol* **45**: 747-754.
Oren A, Ventosa A, Gutierrez MC, Kamekura M (1999). *Haloarcula quadrata* sp. nov., a square, motile archaeon isolated from a brine pool in Sinai (Egypt). *Int J Syst Bacteriol* **49**: 1149-1155.
Pesenti PT, Sikaroodi M, Gillevet PM, Sanchez-Porro C, Ventosa A, Litchfield CD (2008). *Halorubrum californiense* sp. nov., an extreme archaeal halophile isolated from a crystallizer pond at a solar salt plant in California, USA. *Int J Syst Evol Microbiol* **58**: 2710-2715.
Qiu XX, Mou YZ, Zhao ML, Zhang WJ, Han D, Ren M *et al.* (2013). *Halobellus inordinatus* sp. nov., from a marine solar saltern and an inland salt lake of China. *Int J Syst Evol Microbiol* **63**: 3975-3980.
Roh SW, Nam YD, Chang HW, Sung Y, Kim KH, Lee HJ *et al.* (2007a). *Natronococcus jeotgali* sp. nov., a halophilic archaeon isolated from shrimp jeotgal, a traditional fermented seafood from Korea. *Int J Syst Evol Microbiol* **57**: 2129-2131.
Roh SW, Nam YD, Chang HW, Sung Y, Kim KH, Oh HM *et al.* (2007b). *Halalkalicoccus jeotgali* sp. nov., a halophilic archaeon from shrimp jeotgal, a traditional Korean fermented seafood. *Int J Syst Evol Microbiol* **57**: 2296-2298.
Roh SW, Bae JW (2009). *Halorubrum cibi* sp. nov., an extremely halophilic archaeon from salt-fermented seafood. *J Microbiol* **47**: 162-166.
Roh SW, Nam YD, Chang HW, Kim KH, Sung Y, Kim MS *et al.* (2009). *Haloterrigena jeotgali* sp. nov., an extremely halophilic archaeon from salt-fermented food. *Int J Syst Evol Microbiol* **59**: 2359-2363.
Roh SW, Lee ML, Bae JW (2010). *Haladaptatus cibarius* sp. nov., an extremely halophilic archaeon from seafood, and emended description of the genus *Haladaptatus*. *Int J Syst Evol Microbiol* **60**: 1187-1190.
Romano I, Poli A, Finore I, Huertas FJ, Gambacorta A, Pelliccione S *et al.* (2007). *Haloterrigena hispanica* sp. nov., an extremely halophilic archaeon from Fuente de Piedra, southern Spain. *Int J Syst Evol Microbiol* **57**: 1499-1503.
Ruiz-Romero E, Sanchez-Lopez KB, de los Angeles Coutino-Coutino M, Gonzalez-Pozos S, Bello-Lopez JM, Lopez-Ramirez MP *et al.* (2013a). *Natronobacterium texcoconense* sp. nov., a haloalkaliphilic archaeon isolated from soil of a former lake. *Int J Syst Evol Microbiol* **63**: 4163-4166.
Ruiz-Romero E, Valenzuela-Encinas C, Lopez-Ramirez MP, de los Angeles Coutino-Coutino M, Marsch R, Dendooven L (2013b). *Natronorubrum texcoconense* sp. nov., a haloalkaliphilic archaeon isolated from soil of the former lake Texcoco (Mexico). *Arch Microbiol* **195**: 145-151.
Savage KN, Krumholz LR, Oren A, Elshahed MS (2008). *Halosarcina pallida* gen. nov., sp. nov., a halophilic archaeon from a low-salt, sulfide-rich spring. *Int J Syst Evol*

Microbiol **58**: 856-860.
Song HS, Cha IT, Yim KJ, Lee HW, Hyun DW, Lee SJ *et al.* (2014). *Halapricum salinum* gen. nov., sp. nov., an extremely halophilic archaeon isolated from non-purified solar salt. *Antonie Van Leeuwenhoek* **105**: 979-986.
Stan-Lotter H, Pfaffenhuemer M, Legat A, Busse HJ, Radax C, Gruber C (2002). *Halococcus dombrowskii* sp. nov., an archaeal isolate from a Permian alpine salt deposit. *Int J Syst Evol Microbiol* **52**: 1807-1814.
Waino M, Tindall BJ, Ingvorsen K (2000). *Halorhabdus utahensis* gen. nov., sp. nov., an aerobic, extremely halophilic member of the Archaea from Great Salt Lake, Utah. *Int J Syst Evol Microbiol* **50**: 183-190.
Wang QF, Li W, Yang H, Liu YL, Cao HH, Dornmayr-Pfaffenhuemer M *et al.* (2007). *Halococcus qingdaonensis* sp. nov., a halophilic archaeon isolated from a crude sea-salt sample. *Int J Syst Evol Microbiol* **57**: 600-604.
Wang S, Yang Q, Liu ZH, Sun L, Wei D, Zhang JZ *et al.* (2010). *Haloterrigena daqingensis* sp. nov., an extremely haloalkaliphilic archaeon isolated from a saline-alkaline soil. *Int J Syst Evol Microbiol* **60**: 2267-2271.
Xin H, Itoh T, Zhou P, Suzuki K, Kamekura M, Nakase T (2000). *Natrinema versiforme* sp. nov., an extremely halophilic archaeon from Aibi salt lake, Xinjiang, China. *Int J Syst Evol Microbiol* **50**: 1297-1303.
Xin H, Itoh T, Zhou P, Suzuki K, Nakase T (2001). *Natronobacterium nitratireducens* sp. nov., a aloalkaliphilic archaeon isolated from a soda lake in China. *Int J Syst Evol Microbiol* **51**: 1825-1829.
Xu XW, Liu SJ, Tohty D, Oren A, Wu M, Zhou PJ (2005a). *Haloterrigena saccharevitans* sp. nov., an extremely halophilic archaeon from Xin-Jiang, China. *Int J Syst Evol Microbiol* **55**: 2539-2542.
Xu XW, Ren PG, Liu SJ, Wu M, Zhou PJ (2005b). *Natrinema altunense* sp. nov., an extremely halophilic archaeon isolated from a salt lake in Altun Mountain in Xinjiang, China. *Int J Syst Evol Microbiol* **55**: 1311-1314.
Xu XW, Wu M, Zhou PJ, Liu SJ (2005c). *Halobiforma lacisalsi* sp. nov., isolated from a salt lake in China. *Int J Syst Evol Microbiol* **55**: 1949-1952.
Xu XW, Wu YH, Wang CS, Oren A, Zhou PJ, Wu M (2007a). *Haloferax larsenii* sp. nov., an extremely halophilic archaeon from a solar saltern. *Int J Syst Evol Microbiol* **57**: 717-720.
Xu XW, Wu YH, Zhang HB, Wu M (2007b). *Halorubrum arcis* sp. nov., an extremely halophilic archaeon isolated from a saline lake on the Qinghai-Tibet Plateau, China. *Int J Syst Evol Microbiol* **57**: 1069-1072.
Xue Y, Fan H, Ventosa A, Grant WD, Jones BE, Cowan DA *et al.* (2005). *Halalkalicoccus tibetensis* gen. nov., sp. nov., representing a novel genus of haloalkaliphilic archaea. *Int J Syst Evol Microbiol* **55**: 2501-2505.
Yang X, Cui HL (2012). *Halomicrobium zhouii* sp. nov., a halophilic archaeon from a marine solar saltern. *Int J Syst Evol Microbiol* **62**: 1235-1240.
Yang Y, Cui HL, Zhou PJ, Liu SJ (2006). *Halobacterium jilantaiense* sp. nov., a halophilic archaeon isolated from a saline lake in Inner Mongolia, China. *Int J Syst Evol Microbiol* **56**: 2353-2355.
Yang Y, Cui HL, Zhou PJ, Liu SJ (2007). *Haloarcula amylolytica* sp. nov., an extremely halophilic archaeon isolated from Aibi salt lake in Xin-Jiang, China. *Int J Syst Evol Microbiol* **57**: 103-106.
Yim KJ, Cha IT, Lee HW, Song HS, Kim KN, Lee SJ *et al.* (2014a). *Halorubrum halophilum* sp. nov., an extremely halophilic archaeon isolated from a salt-fermented seafood.

Antonie Van Leeuwenhoek **105**: 603-612.

Yim KJ, Cha IT, Whon TW, Lee HW, Song HS, Kim KN *et al.* (2014b). *Halococcus sediminicola* sp. nov., an extremely halophilic archaeon isolated from a marine sediment. *Antonie Van Leeuwenhoek* **105**: 73-79.

Zhang WJ, Han D, Qiu XX, Zhao ML, Mou YZ, Cui HL *et al.* (2013). *Halobellus rarus* sp. nov., a halophilic archaeon from an inland salt lake of China. *Antonie Van Leeuwenhoek* **104**: 377-384.

Zhao ML, Qiu XX, Zhang WJ, Han D, Cui HL, Li ZR (2014). *Halobellus litoreus* sp. nov., a halophilic archaeon isolated from a Chinese marine solar saltern. *Curr Microbiol* **68**: 156-160.
